# How Can We Enhance Patient and Public Involvement and Engagement in Nursing Science?

**DOI:** 10.3390/nursrep15030115

**Published:** 2025-03-20

**Authors:** Richard Gray, Noppamas Pipatpiboon, Daniel Bressington

**Affiliations:** 1 School of Nursing and Midwifery, La Trobe University, Melbourne 3086, Australia; r.gray@latrobe.edu.au; 2 Faculty of Nursing, Chiang Mai University, Chiang Mai 50200, Thailand; noppamas.p@cmu.ac.th

## 1. What Is Patient and Public Involvement and Engagement?

Patient and Public Involvement and Engagement (PPIE, alternatively referred to as public involvement (PI), public and patient involvement (PPI), or consumer and community involvement and engagement (CCIE)), refers to research being conducted ‘with’ or ‘by’ members of the public rather than ‘to’, ‘about’, or ‘for’ them [1]. In this editorial, we have chosen to use PPIE.

The history of patient and public involvement in health research can be traced back to the 1950s and a disillusionment with healthcare decisions being made without consultation with the people impacted by those decisions [2]. Medical scandals—most notably the Alder Hey organ retention scandal of the 1990s where human tissue, including children’s hearts, was retained by the hospital between 1981 and 1996 without consent [3] have given a powerful impetus to a moral imperative to involve the public in healthcare decision making, including research. The shift towards PPIE was also driven by an increasing recognition of the importance of engaging people with ‘lived experience’ when researching a particular health condition, designing clinical services and formulating health and social care policy [4]. The core philosophy of lived experience as a research method is related to phenomenology, where a studied phenomenon is understood from the perspective of how people with lived experience of the phenomena interpret and apply meaning to their existence [5]. The acknowledgement that the relevance of research is likely to be limited without lived experience voices has resulted in an increasing demand for PPIE.

It is worth clarifying what constitutes PPIE and what does not constitute PPIE. PPIE refers to how stakeholders (including patients, families, communities, the public, consumers, caregivers, laypersons, etc.) contribute to a study, whether in the planning, design or conduct of the research, data analysis, interpretation of the results, or dissemination of the findings. PPIE does not refer to participants who only contribute to generating the study’s results, such as completing a survey questionnaire [6] Furthermore, PPIE does not refer to the research team who hold responsibility for the study, unless a member of the team identifies as being an expert by experience (i.e., a patient, carer, etc.). 

## 2. The Need for and Impact of PPIE

Authors have made a powerful moral argument that citizens who pay taxes that fund research have the right to have a voice in how that resource is used. Involving people with lived experience in research ensures that the work addresses the needs of communities and individuals impacted by the research [7]. There is also a case to be made that PPIE enhances the methodological quality, credibility, feasibility, and cost-effectiveness of research (including systematic and other types of literature review) [1,8]. Furthermore, it has been argued that involving patients and other major stakeholders in research enhances decision making processes and improves the relevance of the work for people who use health services [9]. From the patient and public representative’s perspectives, meaningful PPIE can result in gaining knowledge of the healthcare system and how improvements can be made and a feeling of empowerment [10].

## 3. PPIE in Health Research

The case for involving the public in healthcare research is compelling, with many major research funders now requiring the inclusion of information on how the public has been involved in the research [11,12]. This is particularly true in the United Kingdom where the National Institute for Health Research (NIHR), the major funder of health research, requires that in any grant application researchers demonstrate specifically how the public have been involved in the design of the research [13]. Review panels that make funding decisions consist of lay members, and it is widely understood that their views on the merits of the proposed research are taken seriously.

Given the recognized importance of PPIE, there have been several reviews that have sought to determine the extent of public involvement in published research. For example, Price et al. [14] reviewed 152 research papers published over one year in the *BMJ*; 16 (11%) reported some evidence of public involvement, predominantly through the development or selection of outcome measures. Similarly, a recent review [15] of PPIE in Australian randomized controlled trials published in the first six months of 2021 highlighted that even in a country with an advanced healthcare system, authors of only 5% of the 325 reviewed trials reported any public involvement. 

A recent bibliometric analysis [16] of over 10,000 healthcare intervention-related studies published in the Web of Science up to November 2024 highlights that there are global regions where PPIE is limited. Although there has been a considerable increase in the global extent of PPIE reported over the last 5 years, increases in PPIE within Asian countries seem confined to a few settings (i.e., Singapore, Malaysia, and Saudi Arabia), with a conspicuous absence of PPIE work originating from China and some other East Asian countries, such as Thailand [16].

## 4. PPIE in Nursing Research

Nursing as a discipline may have struggled to navigate the aforementioned challenges to embrace PPIE in research. We note, however, that the evidence to substantiate this statement is limited. A descriptive study of the extent of PPIE reported in nursing studies published in leading nursing journals [17] reviewed 89 randomized controlled clinical trials and reported no evidence of public involvement in any of the included studies. However, this bleak picture may be distorted given that nurses often conduct research within multidisciplinary teams and may publish their nurse-led trials in non-nursing journals. Anecdotally, many nurse academics have expressed cynicism about PPIE, citing concerns about tokenism and challenges in establishing and maintaining meaningful engagement. 

*Nursing Reports* strongly supports the use of PPIE and the clear reporting of this involvement in the manuscripts we publish. In March 2023 we introduced an editorial initiative asking authors to include a statement describing how the public had been involved in the research they submit to the journal. This is not a novel initiative; for over a decade the BMJ have required authors of research papers to complete a statement on patient and public involvement in research [18]. We were not even the first nursing journal in nursing to do this—the *Journal of Advanced Nursing* had a similar initiative they launched in 2022 [19], and other Wiley Journals including the *Journal of Clinical Nursing* and *Nursing Open* have followed suit. 

So, why are journal editors (including us) asking authors to report on PPIE? We hoped to achieve three aims: to report on how people with lived experience have been involved in published research in the discipline, to monitor levels of public involvement in nursing science, and to nudge authors to think about involving people with lived experience in their future work. 

According to SCOPUS, in 2024, *Nursing Reports* published 305 documents (papers). It seemed timely to review these manuscripts to determine the level of PPIE reported in these papers. Overwhelmingly, authors reported that there was no PPIE in any aspect of the reported research. The authors of 33 papers (11%) reported public involvement in their research. Authors that indicated involvement of people with lived experience in their research described activities that do not actually constitute PPIE as it is generally accepted. For example, one author mentioned “the expertise and experience of the research team members, with backgrounds in nursing and clinical care, as playing a key role in shaping and implementing the study”. The authors of 24 papers stated that patients or the public had been involved because they took part in the research and completed questionnaires. Statements included “patients took part in the data collection phase of the study” and “nurses were involved by completing a self-administered questionnaire”. To be clear, this is not what is meant by PPIE in research. The issue is not unique to *Nursing Reports*—flick through papers published in the *Journal of Advanced Nursing* or the *Journal of Clinical Nursing* and you will see much the same pattern. Our observation tells us that nurses who do research seem to have a particularly poor understanding of PPIE. For a discipline that likes to think it provides patient-centered care, it is troubling that we are not involving them in the research that we do. Why?

## 5. Why Might Nurse Researchers Struggle to Embrace PPIE?

One likely barrier is that nurse researchers do not know how to involve the public in their work. Other possibilities are that they are unaware of the benefits of PPIE, or that the research environment is not conducive to, or supportive of PPIE. There is little evidence to inform a discussion on this question, as only a few studies have examined nurse researchers’ views of public involvement. One qualitative study exploring the experiences and views of doctoral cancer nurse researchers on PPIE [20] offers some insights. The study involved ten participants from eight countries. The authors reported that despite supporting the general idea, most participants lacked basic knowledge and had received no specific training in involving people with lived experience in their research. The authors also reported that the research environment plays an important role in the researchers’ ability to engage in PPIE, noting that it is promoted and viewed differently in different countries [20]. For example, the junior nurse researchers in countries with emerging healthcare systems (such as Poland and Turkey) reported that PPIE is not viewed as important and consequently there a lack of support and opportunity to engage people with lived experience in their work [20]. 

## 6. How Can We Improve PPIE?

We want to provide some suggestions to readers of *Nursing Reports* about how they can go about involving people with lived experience in their research. These suggestions are based on our observations of principles supporting PPIE whilst recognizing that, as PPIE is contextually bound and culturally mediated, the requirements will vary across different jurisdictions. Many guidelines and checklists provide advice on how to do this, including the European Alliance of Associations for Rheumatology (EULAR) recommendation checklists for patient involvement, which are likely relevant across different clinical settings [21]. Other relevant and perhaps more generic guidelines include the National Survivor Network framework and assessment for measuring and increasing lived experience leadership across the spectrum of engagement [22] and the NHS Health Research Authority best practice guidelines [23]. Australian-based Sydney Health Partners also provide a suite of useful resources to support consumer and community involvement in healthcare design and research [24]. These guidelines are useful, and we recommend reading them.

In our own work, we have co-produced research with people who have had lived experience of mental ill-health or diabetes for many years. We have also conducted studies with a strong PPIE emphasis across diverse cultural groups, particularly those with a traditionally hierarchical view of healthcare professionals and where PPIE recognition and infrastructure may still be developing (i.e., Southeast Asia). Learning from these experiences is useful for other nurse researchers to shape how they engage and work with people with lived experience, as follows. 

## 7. Attitudes Towards PPIE

The researcher’s attitude is a critical factor in the process of PPIE. Effective PPIE cultivates a sense of ownership and shared responsibility among stakeholders, ensuring that the research is meaningful, relevant, and more likely to be translated into clinical practice. Reinforcing the notion of them being an expert by experience has been useful in our earlier work as this illustrates that, without their input, there will be a deficit in the knowledge required to carry out meaningful research.

Facilitating PPIE in research requires effort and hard work. However, equipping public and patient stakeholders with knowledge and understanding, and providing ongoing support, will empower them and foster a positive attitude toward their essential role in enhancing research value, which can substantially contribute to more effective and meaningful engagement. This engagement needs to be early in the research process because the perspectives of nurses on health issues may differ from those of patients or the public; involving patients and the public from the problem assessment stage is essential to accurately capture real-world situations. 

## 8. Recruitment and Payment 

One barrier to involving people with lived experience in research relates to identifying people interested in the work. One practical solution is to advertise in the local community. This can be achieved by displaying posters in hospitals or community health centers (with permission) or via social media posts. Alternatively, charities or other peak bodies related to your field of research can be ideal for identifying people interested in your subject matter. We have used these methods, which have generally been quite successful. 

When you have identified someone interested in becoming involved in your research, the next challenge is finding funding to pay them for their time. Some people will not want payment, but others may. Paying people with lived experience is a complex issue, particularly due to considerations regarding welfare benefits, tax/employment law and payment rates. The UK’s NIHR provide very useful PPIE payment guidance on their website [25] that may be applied to different contexts; however, remember that laws and welfare payment rules vary considerably across different countries. 

It is important that when applying for a research grant, costs associated with PPIE are included. More challenging is finding funding to support public involvement when you are planning a grant submission. Some universities have small funding schemes to support PPIE, but this is the exception and not the rule. That said, most universities will have a central research support office, and it is worth reaching out to ask for support. If you do not ask, you do not get.

## 9. Communication Skills, Including Listening to Feedback

Patients and the public are motivated to help in research—not because of their expert knowledge of research design but because of their real-world experience of what you are studying. You need to explain the research you are proposing using clear, precise language; it requires skilled communication. It is surprising how bad some researchers are at doing this. Most importantly, you need to listen and pay attention to what you are told and embrace the feedback you receive. You should expect your research to change and evolve based on the feedback you are given. This is, after all, the point of PPIE. In our experience, researchers find this challenging. Often having spent years formulating a research idea, it is difficult to change what you planned because someone with lived experience tells you something different to what you previously thought. As an aside, funding bodies are increasingly expecting you to explain how you have changed your research design through the PPIE process; this is one of the key ways in which researchers evidence that they have authentically engaged people with lived experience.

## 10. How to Work with Patients and the Public in Research

People with lived experience can find it stressful and intimidating working with researchers. Good practice is not to invite a patient or member of the public, on their own, to a large research meeting. Ideally, there should be two or more lived experience members in attendance. You should aim to have a pre-meeting where you can go through the agenda and discuss how they might contribute and then have a debriefing session where you can solicit additional feedback and check they were comfortable with how the meeting was facilitated. The chair of the meeting should ensure that they invite lived experience members to the meeting to contribute and acknowledge their participation and feedback to the discussion.

## 11. Ethical Issues

Researchers are often concerned that ethical approval is required to involve patients and the public in their research. Whilst ethics committees may have differing views from country to country, working with people with lived experience does not generally require ethical approval as they are not participants in human research. If you want to check the position in your country, ask your local research ethics committee for guidance; they will be pleased to assist. 

## 12. Conclusions

The involvement of patients and the public in health research is essential for aligning scientific inquiry with real-world health needs and, therefore, improving health outcomes for the communities we serve. Evidence shows that nurse researchers are either reluctant to engage in PPIE, report on it, or perhaps publish it in Nursing Science journals. Here, at *Nursing Reports* we are committed to supporting researchers to involve patients and the public in research from the outset in a meaningful way, and we encourage you to share your PPIE experiences in our journal.
